# An online tool for survival prediction of extrapulmonary small cell carcinoma with random forest

**DOI:** 10.3389/fonc.2023.1166424

**Published:** 2023-06-29

**Authors:** Xin Zhang

**Affiliations:** ^1^ Cancer Center, West China Hospital, Sichuan University, Chengdu, China; ^2^ State Key Laboratory of Biotherapy and Cancer Center, West China Hospital, Collaborative Innovation Center for Biotherapy, Sichuan University, Chengdu, China

**Keywords:** machine learning, SEER database, extrapulmonary small cell carcinoma, survival, online tool

## Abstract

**Purpose:**

Extrapulmonary small cell carcinoma (EPSCC) is rare, and its knowledge is mainly extrapolated from small cell lung carcinoma. Reliable survival prediction tools are lacking.

**Methods:**

A total of 3,921 cases of EPSCC were collected from the Surveillance Epidemiology and End Results (SEER) database, which form the training and internal validation cohorts of the survival prediction model. The endpoint was an overall survival of 0.5–5 years. Internal validation performances of machine learning algorithms were compared, and the best model was selected. External validation (*n* = 68) was performed to evaluate the generalization ability of the selected model.

**Results:**

Among machine learning algorithms, the random forest model performs best on internal validation, whose area under the curve (AUC) is 0.736–0.800. The net benefit is higher than the TNM classification in decision curve analysis. The AUC of this model on the external validation cohort is 0.739–0.811. This model was then deployed online as a free, publicly available prediction tool of EPSCC (http://42.192.80.13:4399/).

**Conclusion:**

This study provides an excellent online survival prediction tool for EPSCC with machine learning and large-scale data. Age, TNM stages, and surgery (including potential performance status information) are the most critical factors for the prediction model.

## Introduction

Small cell carcinoma (SCC) is a poorly differentiated neuroendocrine tumor. SCC mainly involves the lungs, and many studies have drawn reliable conclusions about small cell lung carcinoma (SCLC). Extrapulmonary small cell carcinoma (EPSCC) is much rarer, accounting for 2%–4% of all SCC and 0.1%–0.4% of all cancers (1–3). With limited data on EPSCC accessible, although EPSCC was first described by Duguid and Kennedy in 1930 (4), most of the understanding of the disease was still extrapolated from SCLC (5). However, according to a study of SCC, the incidence of EPSCC continues to increase (annual percent change = 1.58; *p* < 0.05) (1). Thus, focusing more on EPSCC in the future is essential.

EPSCC is aggressive, and the median overall survival (OS) is reported to be only 1.2 years (6). However, since EPSCC is widely distributed throughout the body, the survivals of different organs or systems may have significant differences. For example, the survivals of SCC of the breast are much better than those of the gastrointestinal system. Ochoa et al. reviewed 39 cases of SCC of the breast and reported that OS was 72% at 4 years. Even with stage III EPSCC, 75% of all patients were alive after a median follow-up of 17 months (7). With a relatively small number of studies available, it will be difficult for oncologists to predict the prognoses of EPSCC patients. To date, there are only a few survival analyses of EPSCC. If a prediction model based on large-scale data can be applied in EPSCC throughout the body and has good performance, the problem will be solved.

Thus, the aim of this study is to provide a survival prediction model of EPSCC with multiple-site large-scale data and machine learning algorithms. To further increase the clinical application value of this study, the selected prediction model was deployed online to help physicians evaluate the patients’ survival.

## Method

### Data source

This study is reported using the transparent reporting of a multivariable prediction model for Individual Prognosis or Diagnosis (TRIPOD) statement (8). The checklist can be seen in **Table S1** in **Supplementary Files**.

**Figure 1:**
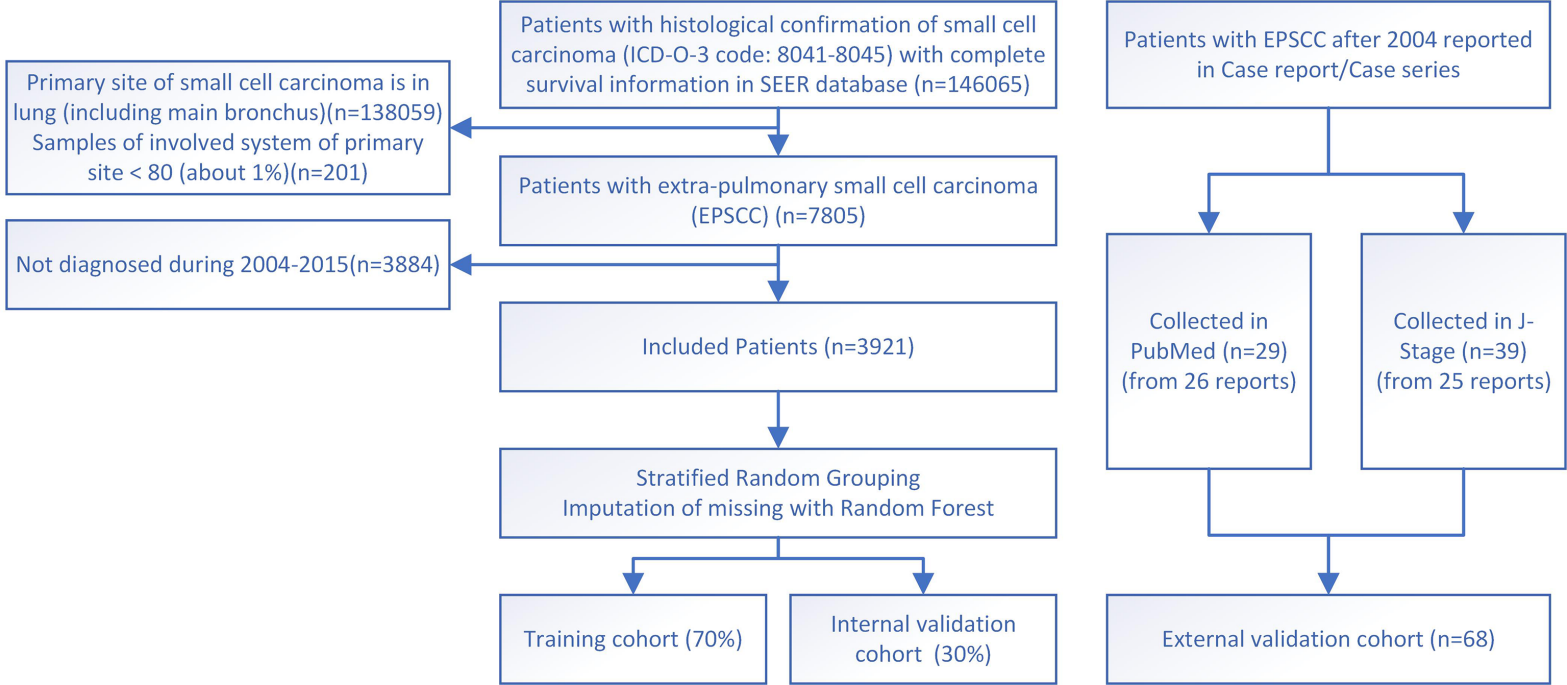
The flowchart for study identification, screening, and inclusion in the training and validation cohort.

Data for the training and internal validation cohort in this study are collected from the Surveillance Epidemiology and End Results (SEER) database (http://seer.cancer.gov/), which contains clinical oncological data from America over 40 years. Data from 17 registries of the United States population during 1975–2019 are downloaded and processed following the steps below. First, the International Classification of Diseases for Oncology, Third Edition (ICD-O-3), histology codes 8041–8045 were used to filter out all small cell carcinoma cases. Then the primary site codes C33–C34 (trachea, bronchus, and lung) were used to exclude SCLC. Other exclusion criteria were as follows: (1) incomplete survival information or follow-up periods ≤ 2 months; (2) multiple primary tumors; (3) the sites are not within the “head and neck”, “urinary system”, “digestive system”, “prostate”, “female genital system”, or “breast”. (There are too few cases of other sites.) Then, the data were screened to those diagnosed during 2004–2015 due to the consistency of the edition of AJCC stage classification and admitted treatment regimen extrapolated from SCLC. Finally, data from 3,921 cases were included in this study as the training and internal validation cohort (**Figure 1**).

External validation data in this study were individual-level data collected from published case reports/series indexed in PubMed or J-Stage. Finally, 68 cases of 51 reports during 2004–2022 were successfully collected and used as the external validation cohort, while other reports do not have survival information (**Figure 1**). The two sources of cases were calculated and analyzed combined and separately, respectively. PubMed-sourced data are based on multiple races, while J-Stage-sourced data are based on an East Asian population different from the SEER cohort, which can prove the generalizability of the prediction model. Details of the external validation cohort are listed in **Table S2** in the **Supplementary Files** (9–39).

### Endpoint and variables

The primary endpoint was overall survival, and the related information is from the “Survival months” and “Vital status recode” variables of SEER.

The collected variables of cases included age, sex, marital status, race, staging, distant metastasis, treatment, and area information. Marital status was divided into three subgroups: married, single, and others (mainly divorced and widowed). “Race and origin recode (NHW, NHB, NHAIAN, NHAPI, Hispanic)” was collected as race information. Staging data include tumor node metastasis (TNM) classification, overall stage and TNM separate stages, and summary stage (*in situ*, localized, regional, and distant). Metastasis data at bone, brain, liver, and lung (“SEER Combined Mets at DX”) were also included. Treatment information includes whether surgery, radiotherapy, or chemotherapy was received. Data about lymph node resection were also collected. Area information means “Median household income inflation adj to 2019” and “Rural-Urban Continuum Code”. In the latter variable, it is regrouped into “Metropolitan”, “Nonmetropolitan”, and “unknown”. Tumor size and extension were not included because of a large amount of missing data. Site-specific factors were also not included since this study involved multiple sites. External validation data were collected after establishing the model. Thus, the reduced variables (race, marital status, and area information) were not collected.

### Establishment of the prediction model

Data preprocessing of this study is all performed on Python (version 3.7.9). According to the limitation of the machine learning algorithm, nominal categorical variables have to be changed to dummy variables. The Random Forest Regressor model was used for imputation. Moreover, the performance of multiple machine learning algorithms was tested. When data standardization was needed, data were scaled in the range of 0–1.

Five machine learning algorithms were explored: random forest, logistic regression, support vector machine (SVM), naïve Bayesian, and XGBoost. The machine learning models were established with “scikit-learn” (version 0.24.1) on Python. The algorithm with the best performance was selected for further calibration, evaluation, and deployment.

Before the adjustment of hyper-parameters, data were stratified—randomly split into the training and internal validation cohorts with a ratio of 7:3. Only training cohort data were used to decide the hyper-parameters. Mean results of 10-fold cross-validation were used to find out the best values of hyper-parameters.

Hyper-parameter of random forest algorithms can prevent overfitting. However, for easier use, feature selection was performed. Area information, race, and marital status were excluded due to less contribution (with low Gini coefficients) to the prediction model. Summary stage, distant metastasis at the liver, and distant metastasis at the brain were excluded because of >0.75 Spearman correlation coefficients with other variables. Thirteen variables were left for the final prediction model.

### Evaluation of the prediction model

Three types of curves were used to evaluate and exhibit the performance of models, which include the receiver operating characteristic (ROC) curve, calibration curve, and decision curve. ROC curve can evaluate the discrimination between alive and dead cases. The area under the ROC curve (AUC) can be compared quantitatively, equal to C-index in the binary classification problem. The calibration curve can evaluate the accordance between predicted survival and actual survival. Finally, the decision curve analysis (DCA) exhibits whether the models provided in this study are better than the TNM staging system. Curves were drawn with Matplotlib on Python.

The training and internal validation cohorts were evaluated for 0.5–5 years. Because of the small sample size of the external validation cohort, there are insufficient data with long follow-up periods. Only 0.5- and 1-year overall survivals of the external validation cohort were tested.

### Interpretability

To gain insight into why the prediction model outperforms the AJCC staging system, the performance of a risk-group classification was explored in this study. The risk groups were evaluated by assessing the percentile of predicted survival probability of each case within the entire external validation cohort. To facilitate comparison with the AJCC staging system whose stages were I–IV, four risk groups were defined. Since the median survival of EPSCC is 1.2 months, as mentioned before, the survival probability used for risk groups was mainly based on the predicted 1-year OS. The Kaplan–Meier (K-M) curve of risk groups was compared with that of AJCC stages. Meanwhile, the random forest model’s feature importance (Gini coefficients) was exported and displayed in a heatmap.

### Deployment

Since the performance of the machine learning model is better than the AJCC staging system, to let this study have a better clinical application value, an interactive website was established with the selected model. Clinical information needs to be entered, and overall survival of 0.5–5 years can be calculated automatically and displayed graphically.

The website is deployed based on Django 2.2.28 (a Python web framework) and elastic computing service is provided by Tencent Cloud company (Shenzhen, China).

### Statistical analysis

SPSS Statistics 26 was used for data description, Kaplan–Meier survival analysis, and Cox regression analysis. Categorical variables were expressed by frequency (*N*) and percentage (%). One-year OS and median survival months were expressed as mean ± standard error or median ± quartile. A 95% confidence interval (CI) of HR of each variable was provided. Student’s *t*-test and the Mann–Whitney *U* test were used for statistical analysis. *p*-value <0.05 was considered statistically significant.

## Results

### Patient characteristics and survival analysis

Patient characteristics of SEER data can be seen in **Table 1**, which is the primary data source of this study. The number of patients with SEER data is 3,912. The total 1-year OS is 43.9% ± 0.8%. The median age is 65–69, and 58.0% are male patients. The most involved sites of SCC in SEER data are the digestive system (30.9%), the urinary system (32.2%), and the female genital system (15.4%). When diagnosed, 43.3% of patients (excluding unstaged) have had distant metastasis, and 47.4% have TNM stage IV. Referring to the treatment of SCLC, most patients have received chemotherapy. However, a large number of patients did not receive surgery or radiotherapy. The training and internal validation cohorts were stratified and randomly divided, and the difference was compared, which is shown in **Table S3** with the characteristics of the external validation cohort (*n* = 68).

**Table 1:** Patient characteristics of SEER data and HR results of Cox regression analyses.

Variable		*N*	%	1-Year OS	HR		*p*-value	
Age	Minors (0–19)	23	0.6%	65.2% ± 9.9%	0.39 (0.23,0.67)		0.001
	Young Adults (20–44)	343	8.7%	62.5% ± 2.6%	0.49 (0.43,0.56)		<0.001
	Middle-aged (45–59)	761	19.4%	52.5% ± 1.8%	0.73 (0.67,0.80)		<0.001
	Elderlies (60+)	2,794	71.3%	39.1% ± 0.9%	Reference		
Gender	Female	1,647	42.0%	46.6% ± 1.2%	Reference		
	Male	2,274	58.0%	42.0% ± 1.0%	1.15 (1.08,1.23)		<0.001
Race	Hispanic	386	9.8%	48.9% ± 2.6%	0.86 (0.76,0.97)		0.014
	White	2,928	74.7%	43.3% ± 0.9%	Reference		
	Black	368	9.4%	44.0% ± 2.6%	1.01 (0.90,1.13)		0.896
	Asian/Pacific Islander	214	5.5%	44.9% ± 3.4%	0.90 (0.77,1.04)		0.161
	Others	25	0.6%	28.0% ± 9.0%	1.64 (1.10,2.45)		0.016
Marital Status	Married	2,369	60.4%	46.7% ± 1.0%	Reference		
	Single	597	15.2%	46.0% ± 2.0%	0.97 (0.88,1.07)		0.584
	Divorced/Widowed/Others	955	24.4%	35.7% ± 1.6%	1.27 (1.18,1.38)		<0.001
Living Area	Metropolitan	3,402	86.8%	44.5% ± 0.9%	0.96 (0.87,1.06)		0.382	
	Un-metropolitan	516	13.2%	40.6% ± 2.2%	Reference		
	Unknown	3	0.1%	44.7% ± 2.4%	7.41 (2.38,23.11)		0.001
Median Household Income (adjusted to 2019)	<$40,000	176	4.5%	36.4% ± 3.6%	Reference		
	$40,000–$70,000	2,318	59.1%	43.2% ± 1.0%	0.93 (0.79,1.09)		0.361
	>$70,000	1,427	36.4%	46.0% ± 1.3%	0.88 (0.75,1.05)		0.154
Site	Digestive	1,213	30.9%	33.0% ± 1.4%	Reference		
	Urinary	1,263	32.2%	45.4% ± 1.4%	0.63 (0.60,0.68)		<0.001
	Female Genital	605	15.4%	53.7% ± 2.0%	0.47 (0.44,0.51)		<0.001
	Male Genital	358	9.1%	36.3% ± 2.5%	0.84 (0.77,0.91)		<0.001
	Head and Neck	380	9.7%	60.0% ± 2.5%	0.50 (0.45,0.55)		<0.001
	Breast	102	2.6%	64.5% ± 4.8%	0.35 (0.30,0.41)		<0.001
TNM Stage	I	405	10.3%	73.7% ± 2.2%	Reference		
	II	663	16.9%	58.4% ± 1.9%	1.47 (1.31,1.65)		<0.001
	III	542	13.8%	57.8% ± 2.1%	1.59 (1.41,1.79)		<0.001
	IV	1,857	47.4%	29.2% ± 1.1%	3.29 (2.98,3.64)		<0.001
	Unstaged	454	11.6%	39.7% ± 2.3%	2.39 (2.15,2.66)		<0.001
Summary Stage	*In situ*	1	0.0%	Not Applicable	Not Applicable		Not Applicable
	Localized	953	24.3%	63.1% ± 1.6%	Reference		
	Regional	994	25.4%	56.1% ± 1.6%	1.30 (1.20,1.41)		<0.001
	Distant	1,698	43.3%	26.5% ± 1.1%	3.00 (2.79,3.22)		<0.001
	Unstaged	275	7.0%	41.0% ± 3.0%	1.95 (1.79,2.12)		<0.001
Surgery	No/unknown	1,903	48.5%	33.4% ± 1.1%	Reference		
	Yes, with LN resection	738	18.8%	64.3% ± 1.8%	0.45 (0.41,0.49)		<0.001
	Yes, no record of LN resection	1,280	32.6%	47.8% ± 1.4%	0.68 (0.64,0.72)		<0.001
Chemotherapy	No/unknown	1,491	38.0%	26.4% ± 1.1%	Reference		
	Yes	2,430	62.0%	54.6% ± 1.0%	0.57 (0.55,0.60)		<0.001
Radiotherapy	No/unknown	2,641	67.4%	36.1% ± 0.9%	Reference		
	Yes	1,280	32.6%	60.0% ± 1.4%	0.61 (0.58,0.65)		<0.001
Total		3,921	100.0%	43.9% ± 0.8%			

HRs of survival were calculated and are listed in **Table 1**. Results show that when compared with the elderlies, the middle-aged have better survival (HR = 0.73, 95% CI 0.67–0.80), and people younger than 45 years old have the best survival (HR = 0.39–0.49). Male (HR = 1.15, 95% CI 1.08–1.23) or “divorced or widowed” (HR = 1.27, 95% CI 1.18–1.38) patients have significantly shorter survival than female or “married or single” patients. Hispanic (HR = 0.86, 95% CI 0.76–0.97) patients have significantly better survival than patients of other races. Among sites, SCC of the digestive system has the worst prognosis. The 1-year OS is 33.0% ± 1.4% and is significantly worse than all other sites. The prognosis of the breast is the best, and its 1-year OS is 64.5% ± 4.8%. All surgery (HR = 0.44–0.66), chemotherapy (HR = 0.52, 95% CI 0.48–0.56), or radiotherapy (HR = 0.61, 95% CI 0.56–0.65) can significantly help prolong survival. It is also seen that with efficient lymph node resection, the HR of surgery (HR = 0.44, 95% CI 0.40–0.48) can be even lower than that of chemotherapy.

### Model evaluation

Machine learning models based on different algorithms were established and tested with the internal validation data. The algorithms and their performance are listed in **Table S4**. Algorithms based on decision trees (random forest and XGBoost) have better AUC than other algorithms, and the AUC of random forest is the best.

**Figure 2:**
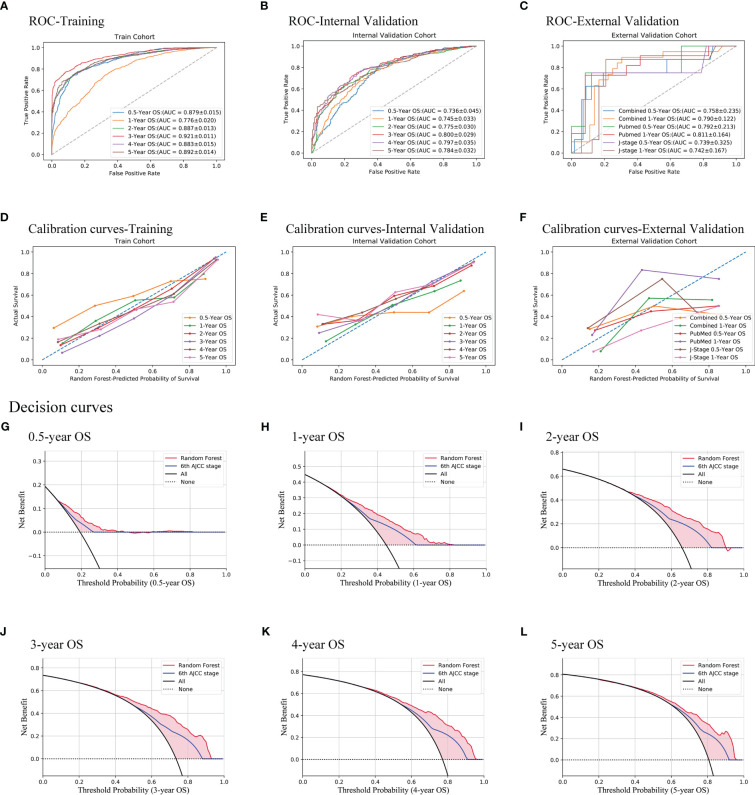
Evaluation of the random forest model. Three methods were used, namely, ROC curves **(A–C)**, calibration curves **(D–F)**, and decision curves **(G–L)**. All of the training cohort **(A, D)**, internal validation cohort **(B, E, G–L)**, and external validation cohort **(C, F)** were used for evaluating the performance of the model. AUCs show good discrimination ability of the model. Decision curves of the internal validation cohort show that the model is better than the AJCC TNM staging system all from 0.5 to 5 years.

After the feature selection and calibration, the random forest model was evaluated with ROC curves, calibration curves, and DCA on all three cohorts (**Figure 2**). The AUCs of 0.5–5 years range from 0.736 to 0.800 and display good discrimination ability (**Figure 2B**). The calibration curves are all near the diagonal (**Figure 2E**), while mean-predicted probabilities are also reliable. Because there are not sufficient external validation data with long follow-up periods, only 0.5- and 1-year overall survivals of the external validation cohort were tested, whose AUCs are 0.758 and 0.790, respectively. Since the data of the external validation cohort were collected from both PubMed and J-stage, the AUCs of different sources were also calculated. The races of J-stage patients are all East Asian, different from that of the SEER database. Thus, the good AUCs of J-stage patients can also be regarded as a justification of generalization ability of this prediction model (**Figure 2C**). **Figures 2G–L** show that the random forest model has a better net benefit than the AJCC staging system in all 0.5- to 5-year survival predictions.

### Interpretability

**Figure 3:**
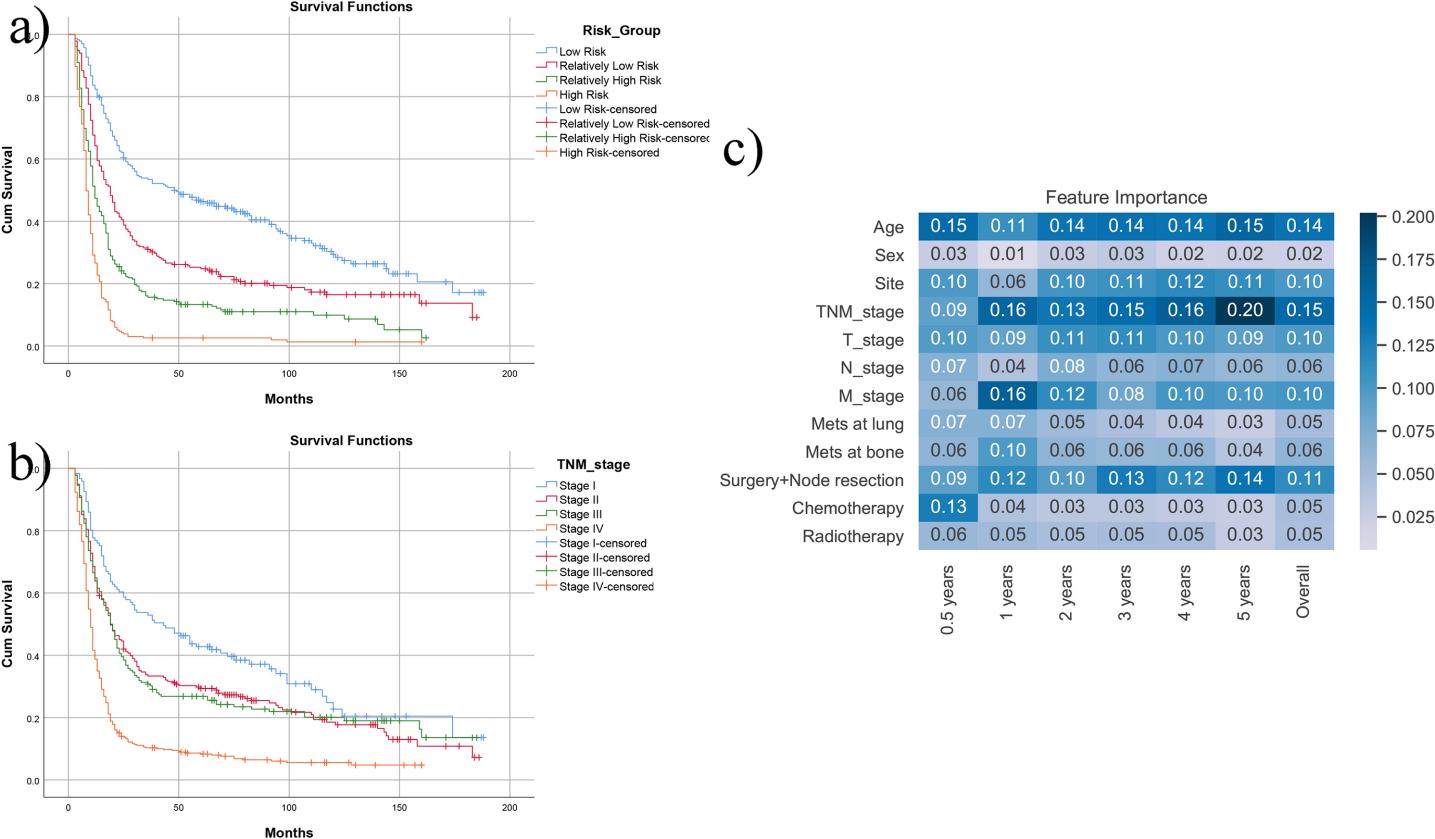
Direct display of the model. **(A)** After dividing the SEER cohort into four risk groups with the predicted probability, it is seen that survival of four groups has apparent difference. **(B)** If using the TNM staging system, stage II and stage III have similar survival. **(C)** The heatmap of the feature importance (Gini coefficient) in the prediction model.

To understand the model more intuitively, patients were divided into four risk groups according to the predicted probability, and the K-M curve is as shown in **Figure 3A**. When compared to the K-M curve of TNM overall stage (**Figure 3B**), it is seen that the model can better divide the cohort because the survival of TNM stages II and III is almost the same.

**Figure 3C** shows the feature importance (Gini coefficients). The higher value and deeper color are more important to the model. Age, sites, overall stage, T stage, M stage, and surgery (including node resection information) considerably influence the prediction of different time lengths. Among them, age, TNM stages, and surgery have the highest importance. Chemotherapy has an enormous influence on 0.5-year survival. However, surgery and node resection (which also includes potential information combined with better performance status and stages) may influence predictions of more extended survival periods.

### Random forest prediction website

**Figure 4:**
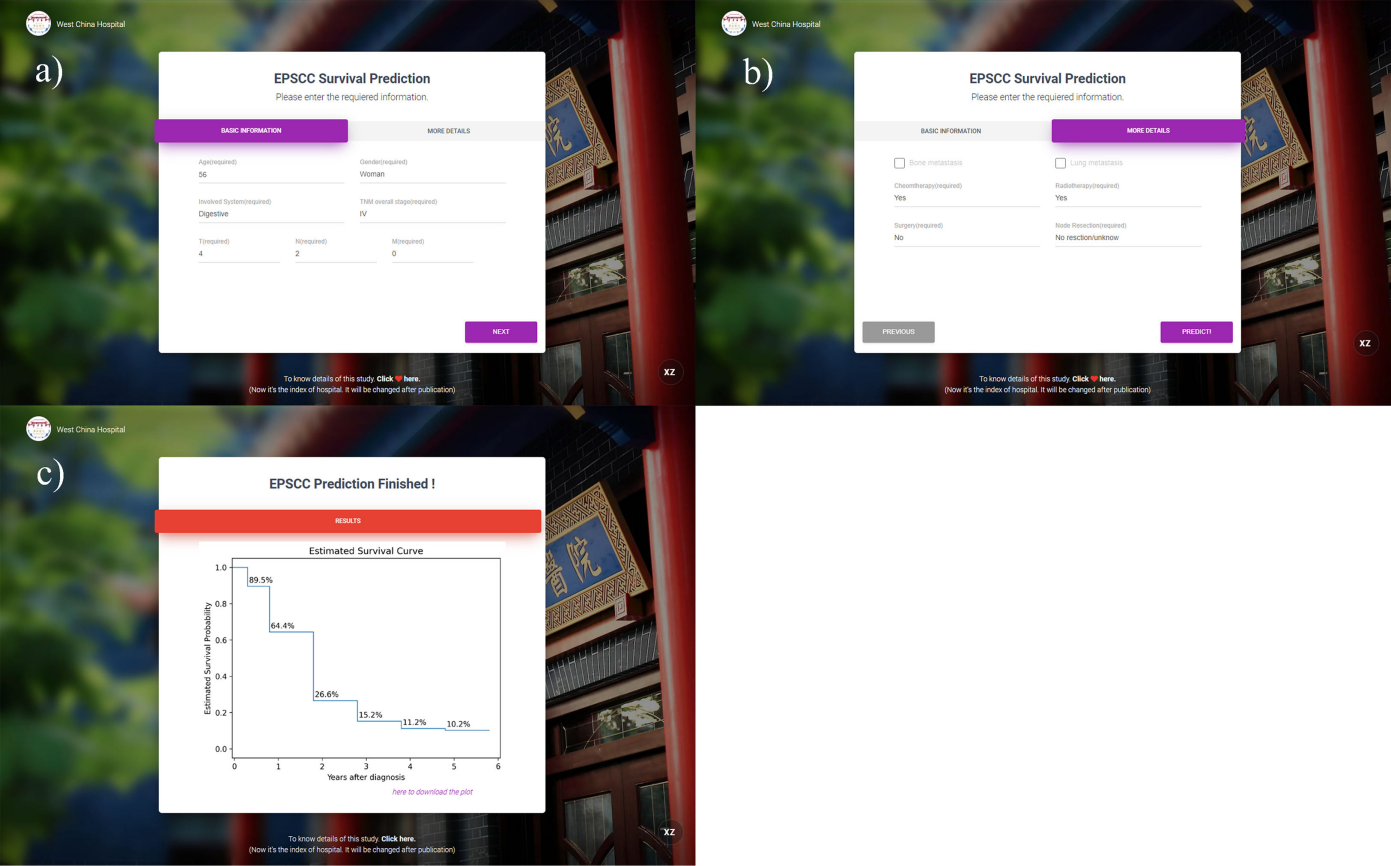
Interface and an example of the prediction website. **(A, B)** The interface for information entering; **(C)** predicted results of 0.5–5 years displayed by the K-M curve (the entered example data: 56-year-old female patient with T4N2M0 overall stage III esophagus EPSCC. Chemotherapy and radiotherapy were received but surgery was not performed).

An interactive online website was deployed on the server. Physicians can access the website *via* http://42.192.80.13:4399/. After entering the required information, prediction result plots will be automatically displayed. Examples can be seen in **Figure 4**.

## Discussion

Despite the increasing incidence of EPSCC (1), the data and research on EPSCC are still limited due to its rarity. Existing studies are mainly case report/series or single-center retrospective analysis. The treatment of EPSCC was extrapolated from that of SCLC or other neuroendocrine tumors since they have similar pathological characteristics. However, different organs have different tumor features, which result in different prognoses. In Canadian data reported by Haider et al., the median OS of gastrointestinal SCC is 4.4 months, while that of breast and gynecology ranges from 40.9 to 54.4 months (40). To date, how long a specific EPSCC patient can survive is also difficult to evaluate. There is still no survival model able to predict EPSCC throughout the body. There are only seven nomograms constructed on single-organ small-scale data (41–47). None of the nomograms have external validation. Thus, this study explores predicting the survival of EPSCC throughout the body with a machine-learning ensemble algorithm and large-scale data. Furthermore, external validation was also performed.

“Machine learning” is the computation process of imitating the human ability to recognize patterns from data and can be used in multiple fields of medicine, regardless of disease diagnosis, prognosis prediction, or screening of potential molecular targets (48). For instance, it was used to predict the diagnosis and prognosis of high-grade B-cell lymphoma with clinical data (49). It was also combined with mRNA expression data to find new biomarkers of adrenocortical tumors (50). The machine learning model’s performance is excellent and constantly increasing with a larger amount of data input. Considering EPSCC is a rare tumor, only extensive databases can filter out a sufficient number of cases. Thus, the SEER database was chosen as the primary data source to compensate for the deficiency of limited data. Since many tumor databases do not contain data on rare tumors, the SEER database and machine learning are the best combination to research rare tumors like EPSCC.

**Table 1** displays the HRs of included factors. It is found that age, sex, race (Hispanic or not), marital status (divorced/widowed or not), primary site, stage, surgery, chemotherapy, and radiotherapy are all independent factors. Younger, female, Hispanic, non-divorced/widowed, early stages, receiving surgery, chemotherapy, and radiotherapy correlate with better prognosis. This is similar to the result provided by Mandish et al., in which sex is insignificant (6). SCCs of the breast and head and neck have better survival, while SCCs of the digestive system and prostate have poor survival in the present study. Similarly, according to Mandish et al., SCCs of the head/neck and breast have a better prognosis, and gastrointestinal SCCs have the worst prognosis (6). It is worth noting that there might be unrevealed prognostic factors because of the lack of specific variables of the SEER database. For example, the ECOG-performance status (PS) score was found to be a good prognostic factor of multiple cancers, especially in elderly patients. However, the PS scores were not included in the SEER and cannot be found to be prognostic factors in this study. However, as a retrospective analysis, the variables of age and whether surgery was received also implied whether the performance status is good or not, considering that surgeons will not perform surgeries on patients with poor PS scores. Thus, the prediction performance is not affected. Additionally, although Hispanic was found to be correlated with better prognosis, it has to be said that the SEER database has a predominantly Caucasian population, whereas other races like African Americans or Asians are underrepresented. Thus, the established prediction model was evaluated with external validation, but further validations with more races are still needed.

In the evaluation of the established prediction model, predicting survivals of multiple-site EPSCCs throughout the body also has good performance (**Figure 2**), compared to the nomograms of seven single-site EPSCC studies, whose AUCs range from 0.656 to 0.75 (41–47). It may be because of the same pathological nature, the large sample size, and the merits of the decision tree-based algorithm. Thus, EPSCC of multiple sites was included in this study to increase the applicable range of the established model. After a comparison of the performance of several machine learning algorithms, it is found that the random forest model performed best on internal validation data. It is reasonable because researchers evaluated 179 classifiers in 121 real-world datasets and found that the random forest is the most likely to be the best (51).

Compared with nomograms, the problem of random forest models is not intuitive. Thus, the Gini coefficients of included factors are given in **Figure 3C**. Age, TNM overall stages, and surgery (including potential performance status information) are the most critical factors for the prediction model, while sex is the one with a minor contribution. Chemotherapy is essential whether the patients can survive within 6 months but may help patients less after 1 year. In the K-M curves of risk groups given by the prediction model (**Figure 3A**), the patients are well divided into subgroups with different prognoses. It performs better than traditional TNM classification (**Figure 3B**), indicating the heterogeneity of stages II and III and its limitations of TNM classification.

Many studies of machine learning prediction models only provide the evaluation results and prove that the method is feasible and reliable. Nevertheless, it is challenging to utilize in clinical work. Thus, to further expand the clinical value of this study, the model was deployed on the online website (http://42.192.80.13:4399/). An example can be seen in **Figure 4**.

Despite its merits, this study still has its limitations. First, it is a retrospective study, which means selective bias is inevitable. Second, EPSCC is a rare type of tumor. Thus, the sample size of the external validation cohort of this study is relatively small. Third, there are some important data in the SEER database, and since EPSCC is rare, it is also not included in most large cancer databases like The Cancer Genome Atlas (TCGA).

Two kinds of variables were lacked in the SEER database. The first one is immunohistochemistry (IHC) staining or molecular test results. For example, Ferro et al. found that Ki-67 <55% indicates poor prognosis but only in metastatic EPSCC with their original data (52) In collecting the external validation cohort, it is found that Chromogranin A, EMA, and TTF1 were commonly stained and the positive rates are in the range 0.60–0.66, which means potential ability for prediction and might be a reference for further investigation. However, there are no IHC data in the SEER database, which limits further analyses. Also, the SEER database does not include variables of tobacco use and performance status. The performance status in the present study can only be indirectly considered with other variables like age and surgery records, which might lessen the accuracy of the analyses. Also, the races in the SEER database should be more balanced. If it is possible to collect current missing variables with the future SEER database or other better population-based tumor databases, this machine learning prediction model must perform much better.

## Conclusions

With large-scale data and machine learning, an excellent prediction model of EPSCC was constructed and deployed online for clinical use. Age, TNM stages, and surgery (including potential performance status information) are the most critical factors for the prediction model.

## Data availability statement

The original contributions presented in the study are included in the article/**Supplementary Material**. Further inquiries can be directed to the corresponding author.

## Ethics statement

This is an observational study based on the SEER public database. No ethical approval, consent to participate, or consent to publish is required.

## Author contributions

The author confirms being the sole contributor of this work and has approved it for publication.
